# Circular Economy Electrochemistry: Creating Additive Manufacturing Feedstocks for Caffeine Detection from Post-Industrial Coffee Pod Waste

**DOI:** 10.1021/acssuschemeng.2c06514

**Published:** 2023-02-06

**Authors:** Evelyn Sigley, Cristiane Kalinke, Robert D. Crapnell, Matthew J. Whittingham, Rhys J. Williams, Edmund M. Keefe, Bruno Campos Janegitz, Juliano Alves Bonacin, Craig E. Banks

**Affiliations:** † Faculty of Science and Engineering, Manchester Metropolitan University, Chester Street, Manchester M1 5GD, United Kingdom; ‡ Institute of Chemistry, University of Campinas (Unicamp), 13083-859 Campinas, Säo Paulo, Brazil; § Department of Nature Sciences, Mathematics, and Education, Federal University of Säo Carlos (UFSCar), 13600-970 Araras, Säo Paulo, Brazil

**Keywords:** additive manufacturing (3D-printing), fused filament fabrication (FFF), fused deposition modeling (FDM), electrochemistry, circular economy, recycling, plastic waste

## Abstract

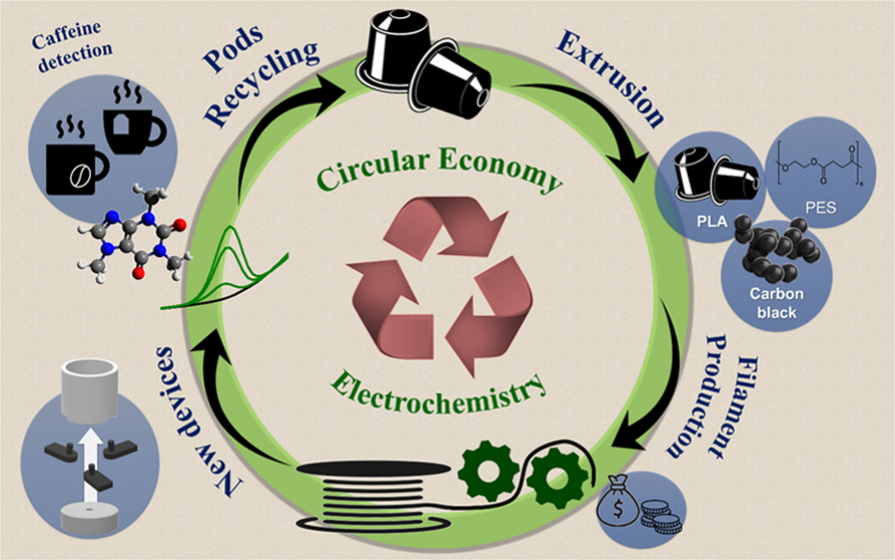

The recycling of post-industrial waste poly(lactic acid) (PI-PLA) from coffee machine pods into electroanalytical sensors for the detection of caffeine in real tea and coffee samples is reported herein. The PI-PLA is transformed into both nonconductive and conductive filaments to produce full electroanalytical cells, including additively manufactured electrodes (AMEs). The electroanalytical cell was designed utilizing separate prints for the cell body and electrodes to increase the recyclability of the system. The cell body made from nonconductive filament was able to be recycled three times before the feedstock-induced print failure. Three bespoke formulations of conductive filament were produced, with the PI-PLA (61.62 wt %), carbon black (CB, 29.60 wt %), and poly(ethylene succinate) (PES, 8.78 wt %) chosen as the most suitable for use due to its equivalent electrochemical performance, lower material cost, and improved thermal stability compared to the filaments with higher PES loading and ability to be printable. It was shown that this system could detect caffeine with a sensitivity of 0.055 ± 0.001 μA μM^–1^, a limit of detection of 0.23 μM, a limit of quantification of 0.76 μM, and a relative standard deviation of 3.14% after activation. Interestingly, the nonactivated 8.78% PES electrodes produced significantly better results in this regard than the activated commercial filament toward the detection of caffeine. The activated 8.78% PES electrode was shown to be able to detect the caffeine content in real and spiked Earl Grey tea and Arabica coffee samples with excellent recoveries (96.7–102%). This work reports a paradigm shift in the way AM, electrochemical research, and sustainability can synergize and feed into part of a circular economy, akin to a circular economy electrochemistry.

## Abstract 2

Recycling of post-industrial coffee pod waste into high-value additive manufacturing feedstocks, introducing circular economy electrochemistry.

## Introduction

The circular economy—although still lacking a formal definition from the International Organization for Standardization—has been a rapidly expanding area of interest within the global scientific community in recent years.^1^ One of the simplest definitions outlined by the Ellen MacArthur Foundation is that “a circular economy is based on the principles of designing out waste and pollution, keeping products and materials in used, and regenerating natural systems”.^2^ Although the aim is to apply this concept to all areas of business and manufacturing, a key area of need is within the production, use, and disposal of plastics.^3^ Plastics have become a staple of modern-day life due to several useful properties. Depending on the plastic type, these properties can include low-cost, chemical resistance, and high specific strength and stiffness. However, the production of “virgin plastics” reliance on nonrenewable energy sources such as oil and the harmful effect persistent waste plastic has on the environment on the natural world are of great concern.^4−6^ Limiting the use of virgin plastics through the recycling of used goods into new products with high value and with an end-of-life plan is a vital component of the circular economy to address. For example, studies have shown that the mechanical recycling of poly(ethylene terephthalate)—another thermoplastic used in additive manufacturing (AM)—can offer an energetic saving of 40–85% over the production of new virgin feedstock.^7^

One area of manufacturing becoming increasingly popular is the use of 3D-printing/AM, which utilizes an additive layer-by-layer approach to manufacturing rather than traditional subtractive or formative methodologies.^8^ The appeal of AM lies in its ability to manufacture complex objects locally and on-demand, which affords a high degree of customizability, reduced waste, and shorter product lead times. Although there are many types of AM, fused filament fabrication (FFF) has seen a surge in interest from both industry and hobbyists due to the sharp reduction in the cost of entry in recent years, with high-performing 3D printers now available for a few hundred pounds or less.^9^ FFF works through the extrusion of a thermoplastic filament through a hot-end and nozzle, which follows a preprogrammed path to produce the desired object; for more information on the printing process, see alternative reviews.^10−13^

Electrochemistry has been one area of research that has seen a dramatic increase in the amount of published research utilizing AM in recent years.^12−14^ In most cases, this is made possible by the inclusion of conductive fillers (commonly carbon) into the thermoplastic filament. Typically, poly(lactic acid) (PLA) is used as the base polymer, and carbon black (CB) and graphene (G) filled commercial filaments are already widely available for purchase worldwide. However, the development of bespoke filaments is being increasingly reported.^15,16^ AM now spans a wide range of electrochemical applications, with many published reports on its use in fuel cells,^17−21^ batteries,^15,22−25^ supercapacitors,^26−29^ and electroanalytical sensing devices.^14,30−33^ The latter began with the printing of simple “lollipop” shape (or disk) working electrodes^34,35^ but has progressed further to electrodes of varying geometries^36^ and the electrochemical cell itself;^37−39^ even accessories^13^ and electrochemical experimental equipment has been reported.^40^ Compared to the use of conventional electrodes, AM allows for the production of electrodes with bespoke geometries at significantly lower manufacturing timescales and costs, by simply altering the computational design.^41,42^

There are significant issues still to address within AM electrochemistry, such as the need to “activate” the surface of the electrode to reveal conductive materials,^34^ surface fouling (as seen with conventional commercial electrodes), and the ingress of solutions into the plastic.^43^ These issues have led to the majority of AM electrodes (AMEs) being single-use items. This makes the printing of electrodes from filament produced through virgin plastic highly unsustainable. Herein, we present a paradigm shift in the production of conductive filaments for electroanalytical applications, using post-industrial waste PLA as the base plastic. The waste PLA used throughout this work originated from coffee machine pods, and we show how this can be turned into a high-grade conductive filament through the addition of CB and a plasticizer, poly(ethylene succinate) (PES), printed to fit in an AM cell (produced from a nonconductive filament of the same origin) and then utilized for the detection of caffeine in both tea and coffee.

## Experimental Section

### Materials

All chemicals used were of analytical grade and used as received without any further purification. All solutions were prepared with deionized water of resistivity not less than 18.2 MΩ cm from a Milli-Q Integral 3 (Merck Millipore, U.K.). Post-industrial waste poly(lactic acid) (PI-PLA), from coffee machine pods, was purchased from Gianeco (Turin, Italy). Hexaamineruthenium (III) chloride (RuHex, 98%), ferrocenemethanol (97%), potassium chloride (>99%), caffeine (99%), sodium hydroxide (>98%), phosphate-buffered saline tablets (pH = 7.4), hydrochloric acid, and poly(ethylene succinate) (PES, MW: 10 000) were purchased from Merck (Gillingham, U.K.). Carbon black (Super P, >99%) was purchased from Fisher Scientific (Loughborough, U.K.). Heat-set inserts were purchased from McMaster-Carr (IL). The commercial conductive PLA/carbon black filament (ProtoPasta, Vancouver, Canada) was purchased from Farnell (Leeds, U.K.). Real samples of Earl Grey Fine Tea (96% Black Tea) and Coffee Bags (100% Arabica Coffee) were purchased from a local convenience store.

### Recycled Filament Production

Prior to mixing, the PLA pellets were dried in an oven at 60 °C for a minimum of 2.5 h to remove any residual water from the polymer. PI-PLA filament was produced by adding the sourced and dried post-industrial waste pellets directly into the hopper of the EX6 extrusion line (Filabot, VA), with the four heat zones set to 60, 190, 195, and 195 °C, respectively. The molten polymer strand was pulled along an Airpath cooling line (Filabot, VA), through an inline measure (Mitutoyo, Japan), and collected on a Filabot spooler (Filabot, VA).

Compositions for the conductive filament were prepared based on a mixing chamber of 63 cm^3^ to produce a filament with PLA (61.62, 59.42, and 57.25 wt %), CB (29.60 wt %), and PES (8.78, 10.98, and 13.15 wt %). The chemicals were blended in a heated chamber (170 °C) with Banbury rotors at 70 rpm for 10 min using a Thermo Haake Poydrive dynameter fitted with a Thermo Haake Rheomix 600 (Thermo Haake, Germany). The resultant sample was allowed to cool to room temperature before being granulated before filament extrusion using a Rapid Granulator 1528 (Rapid, Sweden). This sample was collected and added to the hopper of the EX6 extrusion line (Filabot, VA), with the four heat zones set to 60, 190, 195, and 195 °C, respectively. The molten polymer strand was pulled along an Airpath cooling line (Filabot, VA), through an inline measure (Mitutoyo, Japan), and collected on a Filabot spooler (Filabot, VA).

### Additive Manufacturing

All designs and .3MF files used throughout this work were produced using Autodesk Fusion 360, then sliced and converted to GCODE files using PrusaSlicer (Prusa Research, Prague, Czech Republic). The prints were then produced using FFF technology on a Prusa i3 MK3S+ (Prusa Research, Prague, Czech Republic). The cell body was printed using the PI-PLA filament. Printing parameters for the PI-PLA had a nozzle size of 0.4 mm, temperature of 215 °C, 20% gyroid infill, 3 walls, 0.2 mm layer height, 0.45 mm extrusion width, 1 mm top/bottom thickness, and a 60 mm/s print speed. After printing the heat-set insert was pressed into the printed cavity using a soldering iron. The use of a heat-set insert minimizes the amount of plastic needed to produce a strong threaded connection between cell parts and is easily removable for recycling.

The additively manufactured electrodes (AMEs) were produced both bespoke conductive recycled PLA/carbon black (1.75 mm) and commercial PLA/carbon black (ProtoPasta, 1.75 mm). The parameters for printing each electrode were kept identical, these refer to a nozzle temperature of 215 °C, 100% infill, 3 walls, 0.2 mm layer height, 0.4 mm extrusion width, 1 mm top/bottom thickness, and a 60 mm/s print speed.

### Physiochemical Characterization

Thermogravimetric analysis (TGA) was performed using a Discovery Series SDT 650 controlled by Trios Software (TA Instruments, DA). Samples were mounted in alumina pans (90 μL) and tested using a ramp profile (10 °C min^–1^) from 0 to 800 °C under N_2_ (100 mL min^–1^).

X-ray photoelectron spectroscopy (XPS) data were acquired using an AXIS Supra (Kratos, UK), equipped with a monochromated Al X-ray source (1486.6 eV) operating at 225 W and a hemispherical sector analyzer. It was operated in fixed transmission mode with a pass energy of 160 eV for survey scans and 20 eV for region scans with the collimator operating in slot mode for an analysis area of ∼700 × 300 μm^2^, the FWHM of the Ag 3d5/2 peak using a pass energy of 20 eV was 0.613 eV. Before analysis, each sample was ultrasonicated for 15 min in propan-2-ol and then dried for 2.5 h at 65 °C as this has been shown in our unpublished data to remove excess contamination from PLA and therefore minimize the risk of misleading data. The binding energy scale was calibrated by setting the adventitious sp^3^ C 1s peak to 285.0 eV; this calibration is acknowledged to be flawed,^44^ but was nonetheless used in the absence of reasonable alternatives, and because only limited information was to be inferred from absolute peak positions.

Scanning Electron Microscopy (SEM) measurements were recorded on a Supra 40VP Field Emission (Carl Zeiss Ltd., Cambridge, U.K.) with an average chamber and gun vacuum of 1.3 × 10^–5^ and 1 × 10^–9^ mbar, respectively. Samples were mounted on the aluminum SEM pin stubs (12 mm diameter, Agar Scientific, Essex, U.K,.). To enhance the contrast of these images, a thin layer of Au/Pd (8 V, 30 s) was sputtered onto the electrodes with the SCP7640 from Polaron (Hertfordshire, U.K.) before being placed in the chamber.

### Electrochemical Experiments

An Autolab PGSTAT204 potentiostat (Utrecht, the Netherlands) was used in conjunction with NOVA 2.1.5 (Utrecht, The Netherlands) to carry out electrochemical measurements using a three-electrode configuration. The AMEs were used as the working, counter, and reference electrodes. Where noted, a nickel wire coil was used as the counter electrode and an Ag|AgCl electrode was used as the reference when external electrodes were used. All solutions were prepared using deionized water of resistivity not less than 18.2 MΩ cm from *a* Milli-Q system (Merck, Gillingham, U.K.). Solutions of RuHex were degassed thoroughly for at least 15 min with nitrogen prior to any electrochemical measurement.

Activation of the AMEs, when applicable, was achieved electrochemically in NaOH as described in the literature.^38^ Briefly, the AMEs were connected as the working electrodes in conjunction with a nickel wire coil counter and Ag|AgCl reference electrodes and placed in *a* solution of NaOH (0.5 M). Chronoamperometry was used to activate the AME by applying a set voltage of +1.4 V for 200 s, followed by applying −1.0 V for 200 s. The AMEs were then thoroughly rinsed with deionized water and dried under compressed air before further use.

For real sample analysis, coffee and tea bags were prepared in 250 mL of deionized water for 1 h on a hot plate under stirring.^45^ The samples were then diluted in a 1:10 ratio with the supporting electrolyte (0.1 M phosphate buffer, pH 5.8) for analysis.

## Results and Discussion

Reports of AM being used in electrochemical applications have increased significantly in the last 5 years.^12,13^ Although AM offers a low-waste production methodology, due to its additive nature, sustainability issues are still prevalent due to the use of virgin feedstocks and poor reusability of AMEs. We look to address a key issue in the sustainability of AM in electrochemistry by producing bespoke, high-grade conductive filaments from post-industrial waste and benchmarking their performance against a commercially available alternative; we akin this to circular economy electrochemistry.

### Production and Physical Characterization of Recycled PLA Filaments

**Figure 1:**
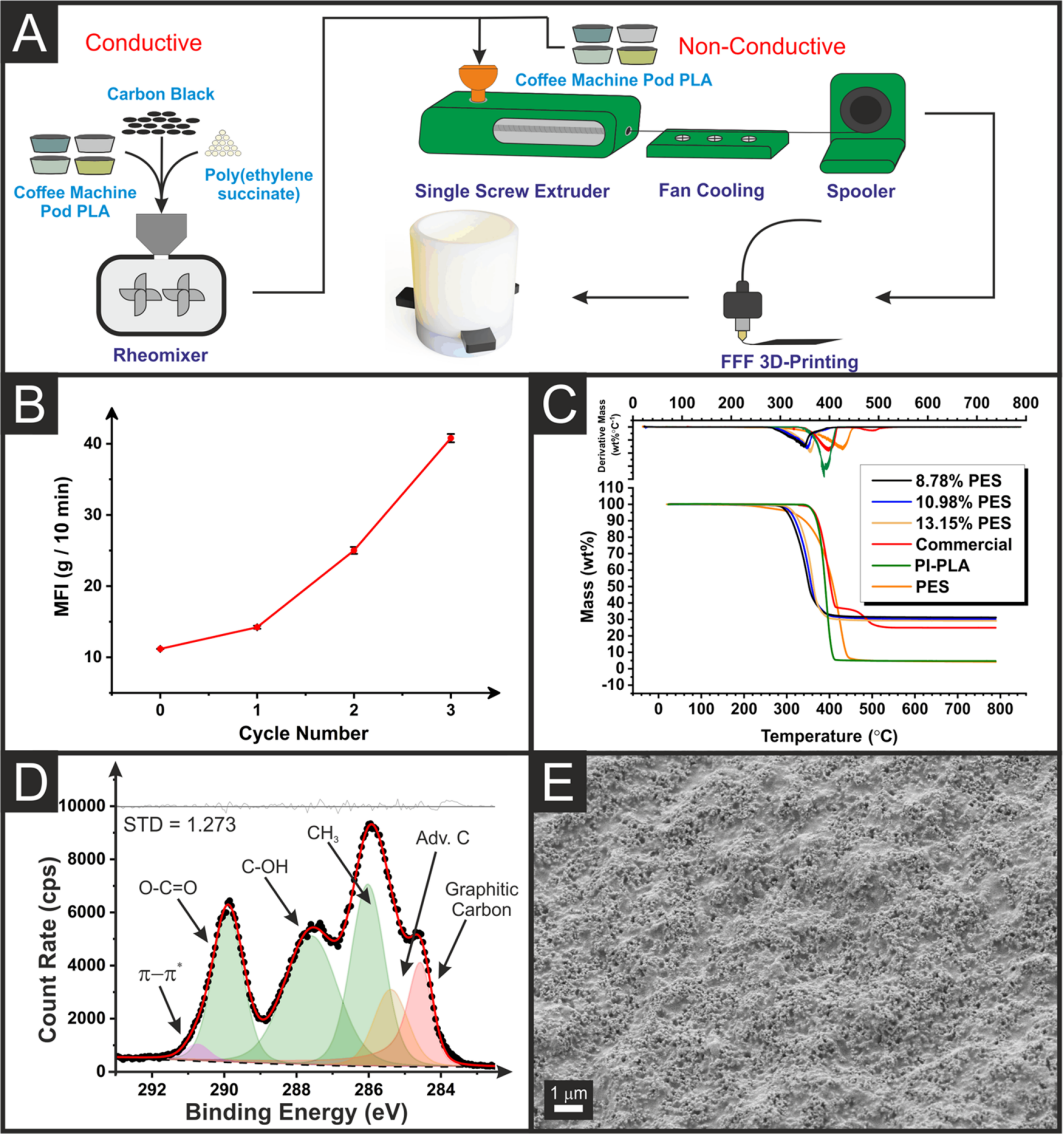
(A) Schematic of the methodology used for bespoke conductive and nonconductive filament production. (B) Melt flow index versus the number of cycles for the PLA cell. (C) Thermogravimetric analysis of bespoke conductive filaments, along with a comparison to the recycled PLA and the commercially sourced conductive PLA filament. (D) XPS C 1s data for the activated 8.78% PES electrode, highlighting the appearance of the graphitic carbon peak after activation. (E) SEM surface image for the activated 8.78% PES electrode.

The PI-PLA used throughout this work was sourced from coffee pods and was dried in the oven for a minimum of 2 h prior to use to remove any residual water from the polymer matrix. In this work, we highlight the production of a nonconductive PLA filament from this material, in addition to three compositions of conductive CB/PLA filaments. The nonconductive filament was produced through the addition of dried, pelletized PI-PLA into the hopper of the extruder and collecting the filament, Figure 1A. This filament was then used to print the electrochemical cell, into which the electrodes could be fixed and sealed. Producing the electrochemical cell and electrodes in two separate prints was chosen to improve the recyclability of the cell, keeping it as a single material rather than mixing in additional additives in the form of carbon black (CB) and poly(ethylene succinate) (PES). To further investigate the recyclability of the electrochemical cell, the production was repeated multiple times using the same feedstock. More specifically, after the printing of the cell, it was repelletized and extruded into a fresh filament, which was then used again to produce the electrochemical cell. This process was repeated for three cycles, where the quality of the print failed to produce a water-tight cell due to the poor quality of the PLA filament obtained after these many cycles from PI-PLA. The degradation of the PLA is shown in the melt flow data, presented in Figure 1B. Melt flow index (MFI) measures the flow of a thermoplastic polymer melt and is an indirect measurement of the molecular weight, whereby an increase in the MFI would indicate a lowering of the molecular weight of the polymeric chains through chain scission caused by thermal and mechanical degradation and a decrease in the viscosity of the polymer melt. In Figure 1B, it can be seen that there is limited change in the melt flow for the initial and first cycle filament, followed by large increases for the subsequent two cycles. This matched visual observations from filament extrusion, whereby each cycle produced a significantly less viscous flow, thought to be caused by polymeric chain scission. Importantly, this highlights how the electrochemical cell can present some failure, be processed, and then recycled into a new filament for the production of a new cell. We note that it may be possible to further enhance the lifespan of this material through the blending of this recycled PLA into a virgin feedstock.^46^

The production of the bespoke conductive filaments is also shown schematically in Figure 1A, where the PI-PLA, CB, and PES were mixed at 170 °C for 10 min, cooled, shredded, and then passed through a single-screw extruder to produce the filament. Three bespoke filaments were produced in this work using different levels of PES (8.78, 10.98, and 13.15 wt %) as the plasticizer, but keeping the amount of CB (29.6 wt %) in the filament constant, this was found to be the highest loading of CB and lowest loading of PES possible while still producing a flexible and printable filament. The bespoke conductive filament (8.78% PES) produced a resistance across 10 cm of filament of 458 ± 15 Ω compared to a quoted value of 2–3 kΩ for the commercial filament.^47^ An interesting observation in the printing profiles of the bespoke and commercial filaments is that the amount of filament required to purge the hot end of the filament is significantly reduced for the bespoke composition, Figure S1A,B. This is attributed to the different plasticizers used in the formulations, leading to the commercial filament sticking in the hot end for longer. This is a useful advantage of the bespoke filament, requiring less purge material means less waste when using multimaterial, single extruder printers. A comparison to other reported conductive filaments in the literature is observed in Table S1, noting this is the first reported using recycled post-industrial waste material. Many of the reports can suffer from poor printability or a brittle filament; the inclusion of PES ensures that the obtained filament has excellent flexibility, Figure S1C,D.

Thermogravimetric analysis of these bespoke filaments is presented in Figure 1C, along with the base PI-PLA and a commercially purchased PLA/CB filament. Analysis of the original feedstock and produced filaments is important to understand whether the historical thermal processing of the PI-PLA and subsequent processing cycles affect the thermal stability of the polymer. Additionally, it can indicate the effect that the plasticizer PES has on the stability of the polymer composite and provide accurate information about the mass of conductive filler present in each formulation. The average onset temperature of each filament, average final mass, and filler contents are presented in Table 1. The CB filler content for the bespoke filaments was calculated by taking the average final mass of the base PI-PLA away from the average final mass of the bespoke filament. In this context, it is assumed any nonpolymeric substance that contributes to the mass remaining in the PI-PLA samples after heating will also be present in the bespoke filament samples after heating. The bespoke filaments were calculated, which had 28 ± 3, 28 ± 3, and 25 ± 5 wt % CB for increasing PES content, an increase in the CB content compared to the commercial filament, calculated to have 21 ± 3 wt % CB which showed good agreement with their technical specification sheet.^48^ Interestingly, as the PES concentration increased in the samples there was a decrease in the average onset temperature from 247 ± 2 to 228 ± 2 °C. It is proposed that the PES is beginning to degrade at a lower temperature, and it blends into the degradation of the PLA as it can be seen that the onset temperature for pure PES is significantly lower than the pure PLA. It has been seen previously that incorporating carbon black into the PLA filament at similar levels does not have a significant effect on the chemistry of decomposition. Indeed, the particles act as physical barriers for gas diffusion out of the polymer, hence serving to slow the rate of decomposition.^49^ The second transition observed in the commercial sample can presumably be attributed to the unknown plasticizer listed by the manufacturer in the material datasheet.^48^

**Table 1:** Thermogravimetric Analysis Results Corresponding to the Post-Industrial Waste PLA, Commercial Conductive Filament, and the Three Bespoke Filaments Produced Using Different Amounts of Poly(ethylene succinate)^a^

filament	average onset *T* (°C)	average final mass (wt %)	filler content (wt %)
base PLA	305 ± 5	3 ± 2	
commercial	304 ± 2	24 ± 2	21 ± 3
PES 8.78%	247 ± 2	31 ± 1	28 ± 3
PES 10.98%	243 ± 3	31 ± 1	28 ± 3
PES 13.15%	228 ± 2	28 ± 4	25 ± 5

a Highlighting the average onset temperature, average final mass, and the conductive filler content of the filament. Uncertainties in values represent the standard deviation obtained from three separate measurements.

To investigate the chemical composition of the bespoke additively manufactured electrodes (AMEs) before (Figure S2) and after activation (Figure 1D) XPS was performed. Before activation, in Figure S2, the C 1s environment shows a spectrum similar to that of PLA, with three peaks of similar intensity corresponding to the three carbon environments in the PLA chain alongside an additional adventitious carbon peak (Adv. C). This suggests that before activation the surface of the electrode is predominantly PLA with the CB particles below the depths probed by XPS (i.e., a few nm).^43^ In contrast, the C 1s spectrum for the activated electrode, Figure 1D, exhibits an additional asymmetric peak at 284.5 eV which is consistent with the X-ray photoelectron emission by graphitic carbon.^50,51^ An additional high-binding energy peak at 290.8 eV is observed for adequate fitting of the activated sample, which arises from π–π* transitions within the graphitic carbon.^50,51^ The presence of this graphitic carbon peak in the activated spectrum provides evidence of the stripping of PLA from the surface and the introduction of CB into the range of XPS. There is an increase in the C–C/C–H peak intensity from the nonactivated to the activated sample. This could also be explained by some PES plasticizer being revealed, as this structure contains a higher ratio of this carbon environment than PLA. It is important to note that after activation and washing of the AME, there was no presence of residual sodium in the wide-angle XPS shown in Figure S2B. Further evidence toward the exposure of CB after activation is seen through the SEM images, in Figures 1E and S3. For the nonactivated 8.78% PES electrode, Figure S3A, there is evidence of carbon particles covered in a polymeric substance, which is substantially removed on the activated sample, as observed in Figure 1E. This removal of PLA can also be seen for the commercial electrode, 10.98% PES, and 13.15% PES samples, Figure S3B–D, respectively. After the bespoke filaments had been physically characterized and shown to be successfully activated, the incorporation into a suitable electroanalytical sensing platform was required.

### Design and Production of the Electrochemical Platform

**Figure 2:**
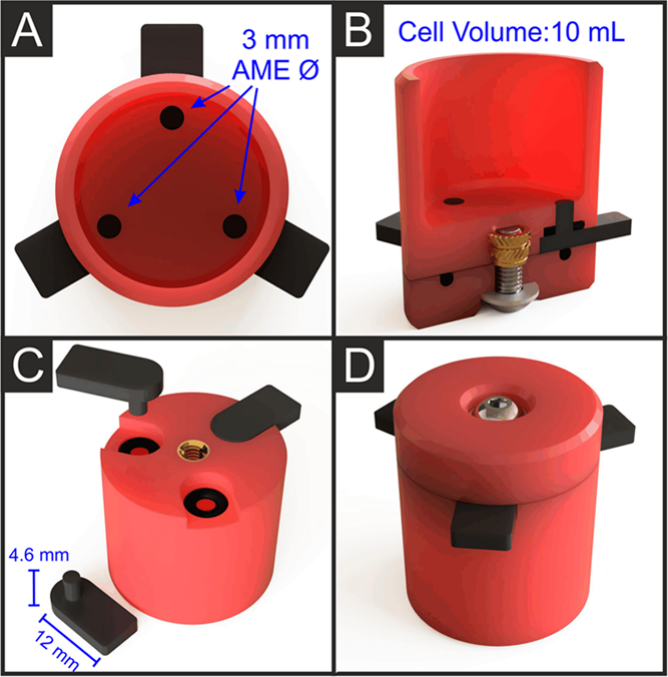
CAD Images of the cell used throughout this work, including (A) top view showing the electrode faces inside the cell, (B) isometric cut-through showing the electrode design, along with bolt, washer, and heat-set insert for sealing the cell, (C) bottom cut-through showing the O-rings and electrode insertion point, along with the heat-set insert, and (D) bottom view showing the bolt for sealing and electrode attachment points.

The design of the electrochemical cell used in this work is presented in Figure 2, with multiple perspectives showing the key aspects of the product. First, the device was split into two separate prints, one using only nonconductive pure PLA filament for the cell, and one using the bespoke conductive filament for the electrodes. It has been shown that embedding the electrodes within the electrochemical cell in a single print is possible and removes the need for external sealing parts.^32^ Otherwise, this introduces significant issues in the recycling process. More specifically, in such devices, the conductive and nonconductive parts cannot easily be separated before processing, and recycling the whole device leads to mixed materials with suboptimal properties. Second, it was important to use all 3D-printed electrodes to reduce the cost of the system as shown in previous work.^32^ Three disk electrodes of identical size (3 mm Ø) are evenly spaced and flush with the bottom of the cell. This allows for the connection of potentiostat leads to any of the electrodes with no change in performance. Figure 2B,C shows the cross section and bottom view of the main body of the cell with electrodes. It can be seen that the electrodes have a thick base for a sturdy connection to crocodile clips, leading into a perpendicular cylindrical section that ends flush with the base of the cell. This cylindrical section allows the addition of O-rings to seal around each electrode through the pressure applied by the baseplate. Additionally, in Figure 2B, the heat-set insert (brass object) can be seen, which is added after the print by simply heating the piece and inserting it into the hole left in the print. This then acts as the thread for the bolt on the bottom piece to attach. Figure 2B,D shows the screw, washer, and baseplate, which provide the necessary force to ensure the cell is water-tight. With this design, the individual pieces can simply be disassembled, the heat-set insert removed, and then the pieces recycled into new products, with the hardware components being cleaned and reused on the next printed cell.

### Electrochemical Characterization of Conductive Recycled PLA (cr-PLA)

**Figure 3:**
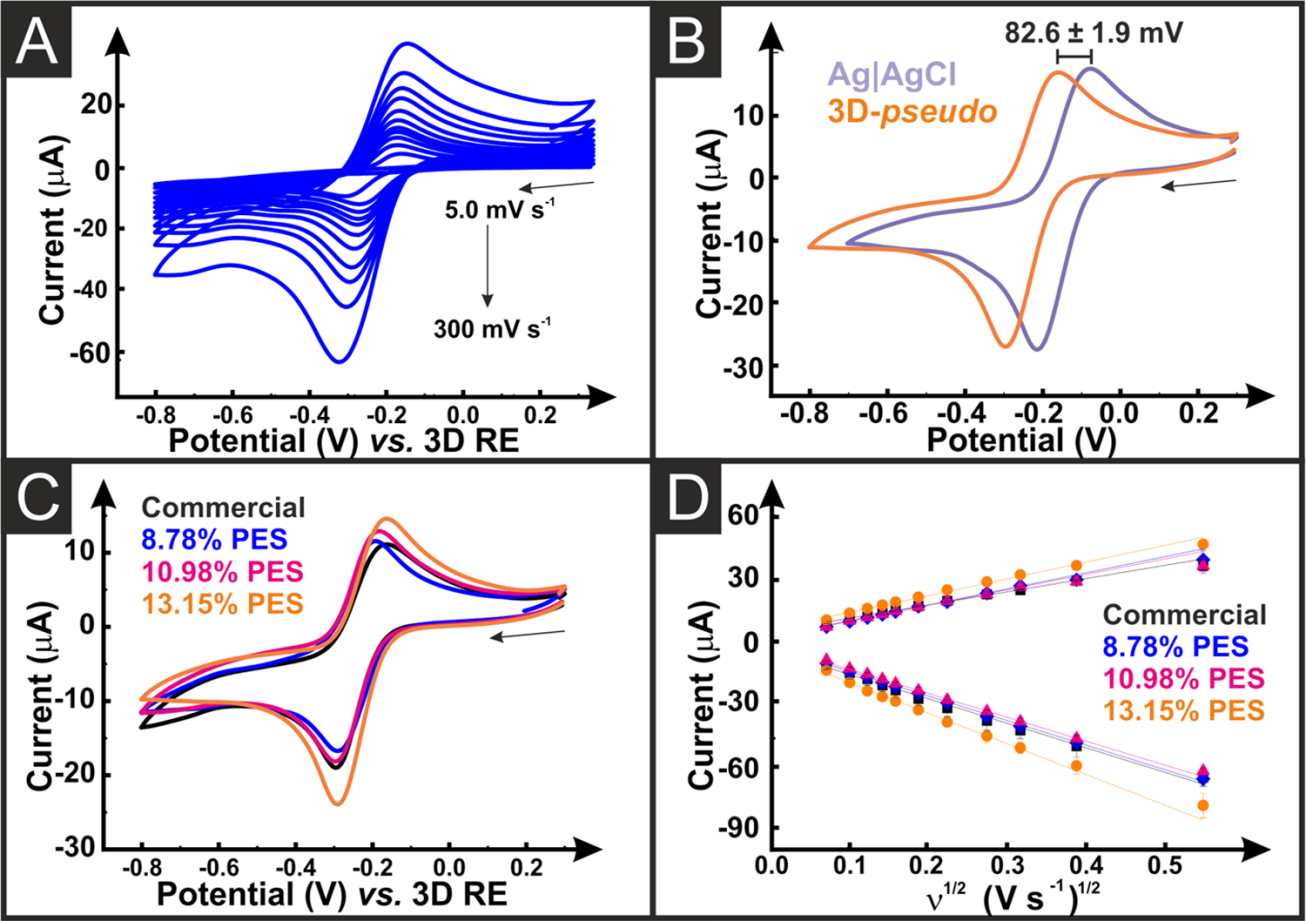
(A) Cyclic voltammograms (5–300 mV s^–1^) of hexaamineruthenium (III) chloride (1 mM in 0.1 M KCl) with the 8.78% PES AME as the working, counter, and pseudo-reference electrodes. (B) Comparison of external and pseudo-reference electrodes for the 13.15% AMEs at 25 mV s^–1^, highlighting the shift in the peak potentials. (C) Overlay of the CVs obtained with AMES printed with the three bespoke filaments and the commercial filament, with 3D-printed pseudo*-*reference and counter electrodes. (D) Randle Sevcik comparison for the AMES printed with the three bespoke filaments and the commercial filament, with 3D-printed pseudo-reference and counter electrodes.

Following the development of an appropriate cell, characterization of the electrochemical performance of the electrodes was performed. Throughout this work, the characterization was performed in two ways, first with a nichrome wire counter electrode and an Ag|AgCl reference electrode as *a* standardized setup as a benchmark, and then second using AMEs as the counter and pseudo*-*reference electrode as this was the intended use in the final electroanalytical platform. Initial electrochemical characterization of nonactivated AMEs was performed using the near-ideal outer sphere redox probe hexaamineruthenium(III) chloride (RuHex).^52^ This allows for the best determination of the heterogeneous electrochemical rate constant (*k*^0^) and the real electrochemical surface area (*A*_e_).^53,54^ This is a far more accurate representation than the geometric surface area on the computer design due to the stratified printing process and unknown surface roughness of the material, especially after activation. A summary of these findings is presented in Table S2. A representation of the scan rate study obtained for a system using all electrodes printed from filament containing 8.78% PES is presented in Figure 3A, with representations for the other tested AMEs presented in Figure S4A–H utilizing both the external and internal counter and reference electrodes. In all cases, the characteristic RuHex wave shape is obtained, with the peaks shifted to more negative values when using the full AM setup, Table S3. This shift is shown for the 8.78% PES AMEs in Figure 3B, whereby a measured shift of 82.6 ± 1.9 mV was observed. However, the peak currents obtained show excellent agreement between the two systems indicating that the use of the fully AM setup is viable. When comparing the AMEs from the four different filaments together, Table S2 and Figures 3C,D and S4, there is no significant difference observed between the electrodes as the calculated *k*^0^ and *A*_e_ are all within error. This indicates that the increased amounts of PES plasticizer were not necessary as the print quality was not noticeably superior as PES content increased, and the cost of PES is significantly higher than the PI-PLA.

**Figure 4:**
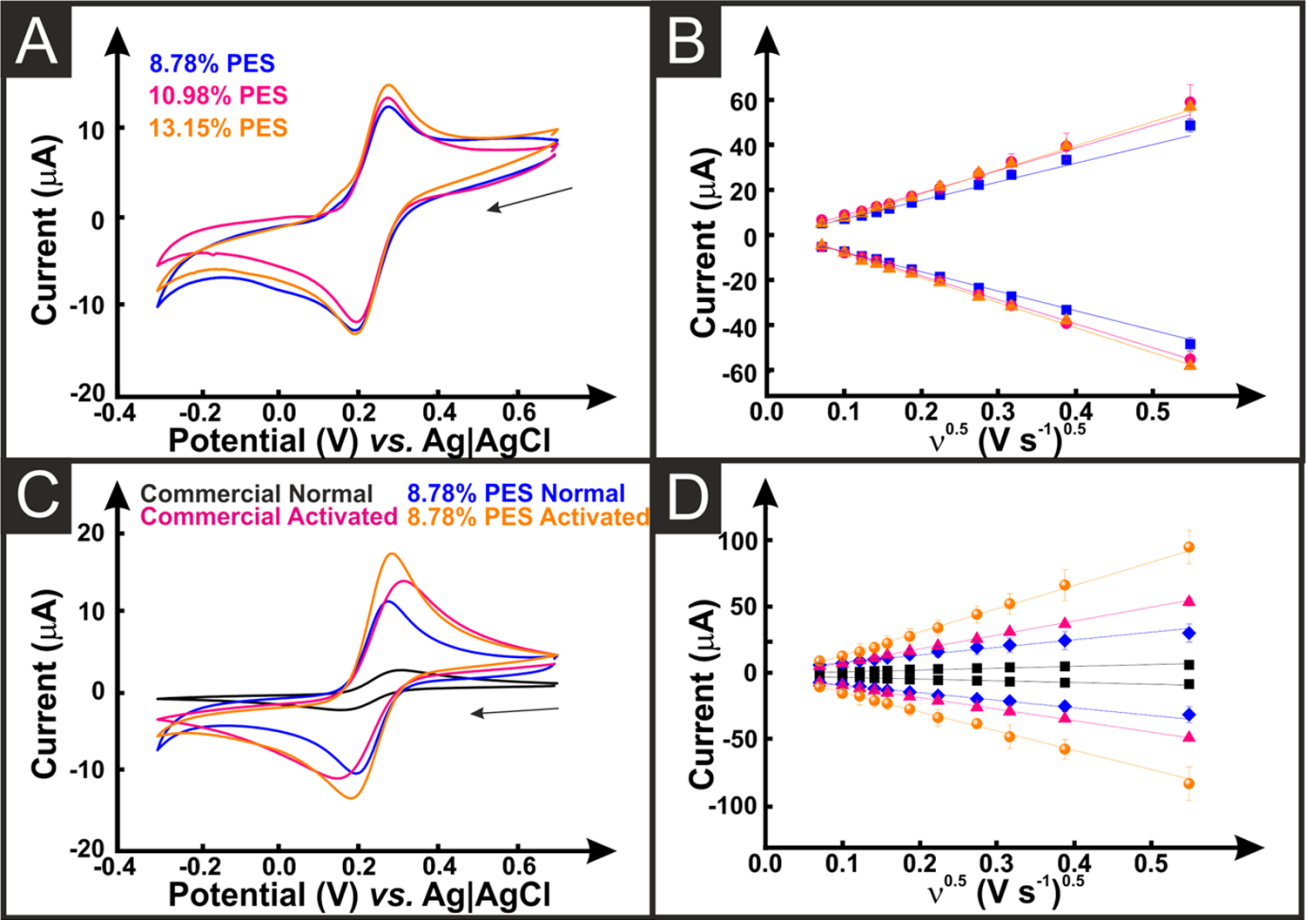
(A) Cyclic voltammograms (25 mV s^–1^) of ferrocenemethanol (1 mM in 0.1 M KCl) with the 8.78, 10.98, and 13.15% PES AMEs as the working electrodes and commercial counter and reference electrodes. (B) Randle Sevcik comparison for the AMES printed with the three bespoke filaments and the commercial filament, with commercial reference and counter electrodes. (C) Cyclic voltammograms (25 mV s^–1^) of ferrocenemethanol (1 mM in 0.1 M KCl) with the nonactivated and activated 8.78% PES and commercial AMEs as the working electrodes and commercial counter and reference electrodes. (D) Randle Sevcik comparison for the AMES printed with nonactivated and activated 8.78% PES and commercial AMEs as the working electrodes with commercial reference and counter electrodes.

As such, we moved to characterize the electrodes using the common inner-sphere probe ferrocenemethanol, Figure S5, for both nonactivated and activated AMEs produced from all four filaments. The peak-to-peak separation (Δ*E*_p_), *k*^0^, and *A*_e_ are presented in Table 2, where it can be seen that in all cases of the bespoke filaments, there is a significant decrease in Δ*E*_p_ and an increase in the calculated *k*^0^ and *A*_e_, indicating activation of the electrodes produces an improved electrochemical performance regarding this inner-sphere probe. When comparing the performance of the electrodes, all three bespoke electrodes offer significant improvements over electrodes made from the commercial filament, although there are no significant differences seen between the three bespoke filaments (Figure 4A,B) once again indicating that the increased levels of PES plasticizer were redundant. It was once again observed that when switching between commercial to AM counter and reference electrodes, a shift was observed to more negative peak potentials by ∼130 mV, as observed in Figure S6. In all previous reports of AMEs for electroanalytical purposes, activation has been key to producing good performance with commercially purchased filament. Curiously, when comparing the improvement in electrochemical response to ferrocenemethanol for both the commercial filament and 8.78% PES bespoke filament a much larger percentage improvement in peak current is seen for the commercial filament after activation. Otherwise, the bespoke filament is capable of producing results comparable to the activated commercial filament prior to activation (Figure 4C,D), highlighting the quality of the bespoke filament. Having characterized the electrochemical properties of the filaments in this work, the 8.78% PES filament was chosen for the electroanalytical studies as all of the bespoke filaments provided similar performance, with the 8.78% filament being the lowest in material cost (due to containing the lowest amount of PES) and producing the best thermal stability.

**Table 2:** Comparisons of the Calculated Peak-to-Peak Separations (Δ*E*_p_), Heterogeneous Electron Transfer (*k*_obs_^0^), and Electrochemically Active Area (*A*_e_) for the Filaments Used in This Work^a^

	filament	Δ*E*_p_ (V)	*k*_obs_^0^ (cm s^–1^)	*A*_e_ (cm^2^)
nonactivated	C-PLA/CB	0.157 ± 0.038	(1.03 ± 0.31) × 10^–3^	0.019 ± 0.007
	8.78% PES	0.102 ± 0.020	(2.05 ± 0.82) × 10^–3^	0.079 ± 0.002
	10.98% PES	0.105 ± 0.021	(1.99 ± 0.79) × 10^–3^	0.070 ± 0.002
	13.15% PES	0.100 ± 0.021	(2.11 ± 0.85) × 10^–3^	0.074 ± 0.005
NaOH + EChem activated	C-PLA/CB	0.160 ± 0.029	(1.10 ± 0.25) × 10^–3^	0.084 ± 0.001
	8.78% PES	0.085 ± 0.016	(2.48 ± 1.08) × 10^–3^	0.130 ± 0.003
	10.98% PES	0.084 ± 0.019	(2.45 ± 1.00) × 10^–3^	0.147 ± 0.006
	13.15% PES	0.086 ± 0.017	(2.44 ± 1.07) × 10^–3^	0.155 ± 0.004

a Calculated using cyclic voltammetry (5–300 mV s^–1^) in a solution of ferrocenemethanol (1.0 mM in 0.10 M KCl), with a nichrome wire counter electrode and Ag|AgCl reference electrode.

### Electroanalytical Determination of Caffeine

**Figure 5:**
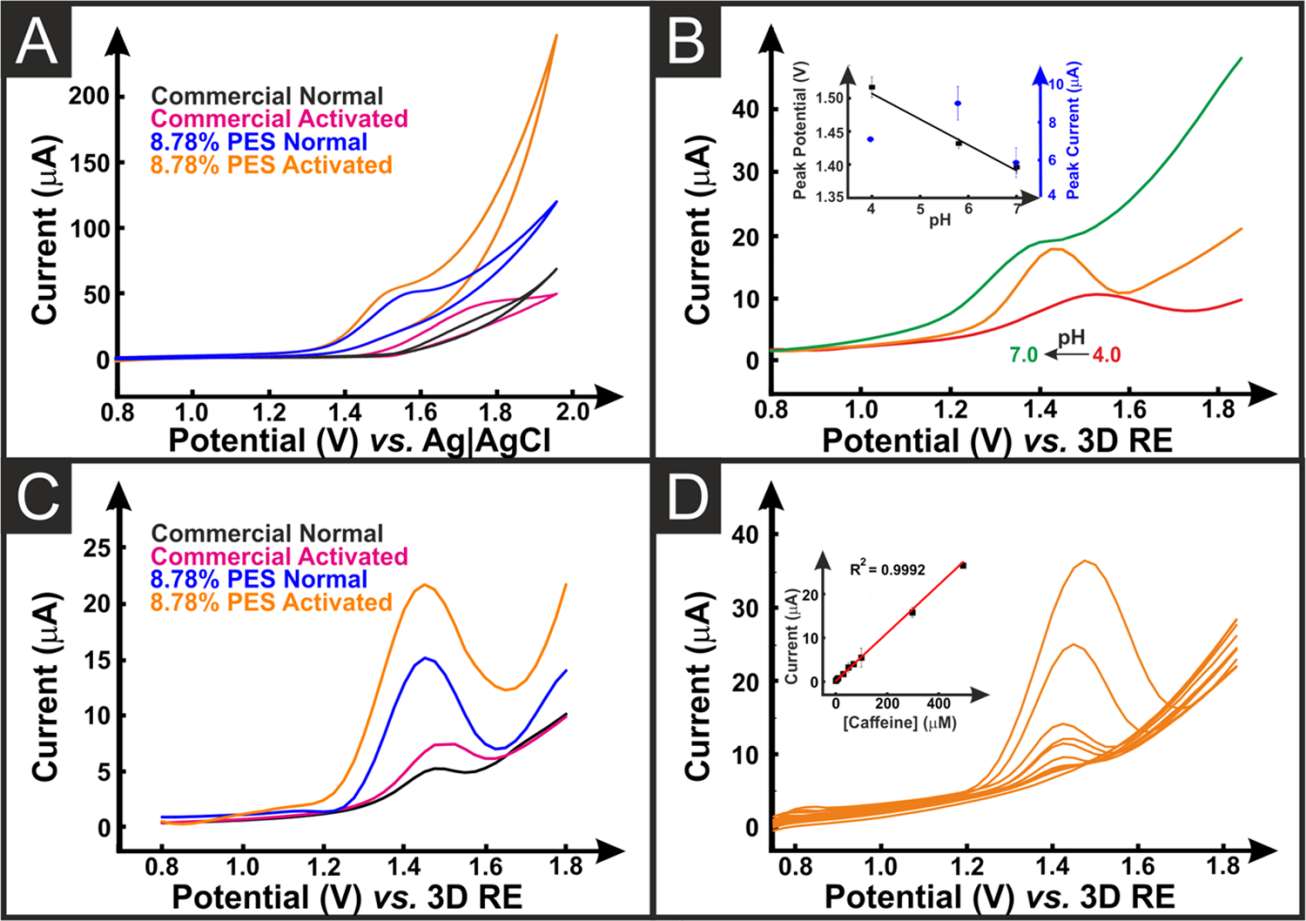
(A) Cyclic voltammograms (25 mV s^–1^) of caffeine (100 μM in 0.10 M phosphate buffer) with nonactivated and activated 8.78% PES and commercial AMEs as the working electrodes and AM counter and pseudo-reference electrodes. (B) Differential pulse voltammograms of caffeine (100 μM at pH 4.0, 5.8, and 7.0) with activated 8.78% PES AMEs as the working electrodes and AM counter and pseudo-reference electrodes. Inset is a plot of the peak current and peak potentials. (C) Differential pulse voltammograms of caffeine (100 μM at pH 5.8) with nonactivated and activated 8.78% PES and commercial AMEs as the working electrodes and AM counter and pseudo-reference electrodes. (D) Differential pulse voltammograms of caffeine (pH 5.8) with activated 8.78% PES AMEs as the working electrodes and AM counter and pseudo-reference electrodes. Inset is a plot of the peak current versus the concentration of caffeine.

To complete a serendipitous circle for the PI-PLA, sourced from coffee machine pods, the electroanalytical cell was utilized for the detection of caffeine in coffee and tea. First, to check the possibility of this work, the potential window for the electrodes was tested, Figure S7A. This showed that the bespoke filaments could be used past +1.5 V versus an AM pseudo*-*reference electrode. Cyclic voltammograms for 100 μM caffeine in 0.10 M phosphate buffer using activated and nonactivated AMEs printed from commercial, and 8.78% PES filament are presented in Figure 5A. An anodic peak was recorded, attributed to the irreversible oxidation reaction of caffeine to uric acid-4,5 diol, Figure S8. The peak was able to be obtained in all cases, with the bespoke filament producing peaks at lower potentials and increased peak currents. Interestingly, the nonactivated 8.78% PES electrode produced a significantly improved voltammogram over the commercial-activated filament: indicated by the shift to less positive peak potentials, a clearer wave shape, and an increased peak current. To optimize the system for the detection of caffeine, the performance was checked at different pH values (4.0, 5.8, and 7.0), Figure 5B. There was a shift to more negative values with decreasing pH and the highest peak current obtained was at pH 5.8, which was chosen for further use. A comparison of the differential pulse voltammetry (DPV) response for the nonactivated and activated electrodes produced from commercial and 8.78% PES filament in the presence of 300 μM caffeine is presented in Figure 5C, which shows significantly improved performance from both the bespoke electrodes compared to the commercial. In both cases, there is an improvement when utilizing the activated over the nonactivated electrodes, but once again the nonactivated 8.78% PES electrode produces an enhanced performance over the activated commercial electrodes. This would allow for an electroanalytical system to be produced straight from the print bed, requiring no further treatment, which is a large step forward in combining AM with electroanalytical sensing. Figure 5D shows the linear DPV response (*R*^2^ = 0.9992) of the activated 8.78% PES filament and analytical linear curve inset, following the equation: *I*_pa_ (μA) = 0.0548(0.0005) *C*_CAF_ μmol L^–1^ + 0.153(0.008), with the others presented in Figure S7B–D. The data obtained from these plots are summarized in Table 3, where the 8.78% PES filament produced a wider linear detection range, of 1.0–500 μM (1.0, 5.0, 10, 50, 70, 100, 300, and 500 μM), and improved limits of detection and quantification (LOD, LOQ), calculated through the 3σ and 10σ methodologies. The best-performing system was found to be the activated 8.78% PES electrode with a sensitivity of 0.055 ± 0.001 μA μM^–1^, a LOD of 0.23 μM, a LOQ of 0.76 μM, and a relative standard deviation (RSD) of 3.14%, Figure S9. The activated 8.78% PES system was then tested for the detection of caffeine in real Earl Grey Tea and Coffee samples, Table 4, with excellent recovery values (96.7–102%) found in all cases, which highlights the impressive performance of these electrodes. A comparison of the system characterized here and other reported electroanalytical devices for caffeine detection can be seen in Table S4, highlighting the performance of this platform produced from recycled PI-PLA.

**Table 3:** Comparison of the Electroanalytical Detection of Caffeine in Phosphate Buffer (pH = 5.8) for the Commercial PLA/CB Filament and the Recycled Filaments with 8.78% PES, Both Activated and Nonactivated^a^

	electrode	LDR (μM)	sensitivity (μA μM^–1^)	LOD (μM)	LOQ (μM)	RSD (%)
nonactivated	C-PLA/CB	50–300	0.036 ± 0.001	14.6	48.7	4.37
	8.78% PES	1.0–500	0.043 ± 0.001	0.27	0.88	3.60
NaOH + EChem activated	C-PLA/CB	50–300	0.039 ± 0.001	13.4	44.6	3.48
	8.78% PES	1.0–500	0.055 ± 0.001	0.23	0.76	3.14

a Highlighting the linear dynamic range (LDR), sensitivity, limit of detection (LOD), limit of quantification (LOQ), and relative standard deviation (RSD).

**Table 4:** Caffeine Levels Found in Real (Top Line for Each) and Spiked Tea and Coffee Samples (*n* = 3) Using the Activated 8.78% PES Recycled Filament

	caffeine added (M)	caffeine found (M)	recovery (%)
Earl Grey Tea		2.59 × 10^–6^	
	3.00 × 10^–6^	2.95 × 10^–6^	98.5
	5.00 × 10^–6^	5.14 × 10^–6^	103
	1.00 × 10^–6^	9.88 × 10^–6^	98.8
Arabica Coffee		1.50 × 10^–5^	
	2.00 × 10^–5^	2.02 × 10^–5^	101
	3.00 × 10^–5^	2.90 × 10^–5^	96.7
	1.00 × 10^–4^	1.02 × 10^–4^	102

This work represents a step change in the way additive manufacturing and electrochemistry can synergize with the circular economy concept. In addition, our work follows the UN Sustainable Development Goals (SDGs), in particular Goal 12—Responsible Consumption and Production, aiming to raise awareness about the recycling of plastic waste for the production of new materials and products.^55^ It highlights how post-industrial plastic waste can be utilized to produce electrodes capable of significantly improved performance over current commercially available conductive filament. We hope these results spark further research into improving the sustainability of electrochemical research and product development; we hope that others join to expand circular economy electrochemistry.

## Conclusions

This work describes the production of bespoke additive manufacturing feedstock from post-industrial plastic waste poly(lactic acid). Both nonconductive and conductive filaments are produced and physiochemically characterized. A bespoke electrochemical device was designed and produced in separate prints allowing for the simple recycling of the components (in comparison to all-in-one printed devices). The nonconductive filament was able to be cycled through use three times before print failure, allowing the body of the electrochemical cell to be used to product failure and then repurposed. Three bespoke filaments were produced with carbon black as the conductive filler (29.6 wt %) and poly(ethylene succinate) as the plasticizer (8.78, 10.98, and 13.15 wt %). These were characterized electrochemically using both inner and outer sphere redox probes and benchmarked against a commercially available conductive filament. All bespoke filaments exhibited similar characteristics, including significantly enhanced electrochemical performance over the commercially purchased one, which indicated that the increased CB filler enhanced the performance but increasing the PES plasticizer from 8.78 to 13.15 wt % did not. The 8.78% PES filament was found to be the most suitable for the end product due to its reduced material cost (due to the lower PES content) and increased thermal stability. This filament was found to detect caffeine with a sensitivity of 0.055 ± 0.001 μA μM^–1^, a LOD of 0.23 μM, a LOQ of 0.76 μM, and an RSD of 3.14% after activation. Interestingly, the nonactivated 8.78% PES electrodes produced significantly better results than the activated commercial filament toward the detection of caffeine. The activated 8.78% PES electrode was shown to be able to detect the caffeine content in real Earl Grey Tea and Coffee samples with excellent recoveries. This work reports a paradigm shift in the way additive manufacturing and electrochemical research can be performed sustainably and form part of a circular economy electrochemistry. We hope future research continues to push boundaries in this area through exploration of different recycled plastics, use of different plasticizers, and maximizing conductive filler efficiency.

## Supporting Information Available

Comparison tables of bespoke filament compositions and alternative caffeine sensors in the literature; tables of data corresponding to the scan rate studies and reference electrode testing; photographs of purging tests and filament flexibility; SEM images for different compositions of AME; XPS for a nonactivated electrode; scan rate study and reference testing voltammograms; potential window testing and analytical curves for alternative filaments; reaction mechanism of the oxidation of caffeine; and reproducibility studies (PDF)
