# Supplementary material for: Circular Economy Electrochemistry: Creating Additive Manufacturing Feedstocks for Caffeine Detection from Post-Industrial Coffee Pod Waste
Source: ACS Sustain Chem Eng. 2023 Feb 6;11(7):2978–88. doi: 10.1021/acssuschemeng.2c06514 (PMC9945317; doi:10.1021/acssuschemeng.2c06514)
Supplement: Supplementary file 1 — sc2c06514_si_001.pdf [file sc2c06514_si_001.pdf]

Supporting Information for:

Circular economy electrochemistry: Creating additive manufacturing feedstocks for caffeine detection from post-industrial coffee pod waste

Evelyn Sigley,[a] Cristiane Kalinke,[a,b] Robert D. Crapnell,[a] Matthew J. Whittingham,[a] Rhys J. Williams,[a] Edmund M. Keefe,[a] Bruno Campos Janegitz,[c] Juliano Alves. Bonacin[b] and Craig E. Banks[a*]

[a]: *Faculty of Science and Engineering, Manchester Metropolitan University, Chester Street, M1 5GD, United Kingdom.*

[b]: *Institute of Chemistry, University of Campinas (Unicamp), 13083-859, São Paulo, Brazil*

[c]: *Department of Nature Sciences, Mathematics, and Education, Federal University of São Carlos (UFSCar), 13600-970 Araras, São Paulo, Brazil*

[*]To whom correspondence should be addressed.

E-mail: c.banks@mmu.ac.uk; Tel: +44(0)1612471196

Content includes: Pages: 11; **Figures**: 8; **Tables**: 4

Table S1. Comparison of the composition of the filaments in this work to other bespoke conductive filaments found in the literature.

**Conductive Material (wt%)**	**Base Polymer**	**Additional Materials**	**Application**	**Reference**
Graphene (20%)	PLA	-	Li-Ion Battery	[1]
Graphite (40%)	PLA	-	SARS-CoV-2 detection	[2]
Carbon Black (32.3%)	PP	-	Electrical circuits	[3]
Activated Charcoal (22.7%)	PLA	MoS_2/PEG/MWCNT	Energy Storage	[4]
Carbon Black (20%)	ABS	PVA	Na-Ion Battery	[5]
Nanographite (25%)	PLA	-	Heavy metal detection	[6]
Carbon Black (15%)	PLA	2D-$MoSe_2$	Water splitting	[7]
Carbon Black (29.6%)	*Recycled PLA*	*PES*	*Caffeine detection*	*This Work*

Key: PLA: poly(lactic acid); PP: poly(propylene); ABS: poly(acrylonitrile butadiene styrene); PVA: poly(vinyl acrylate); PEG: poly(ethylene glycol); MWCNT: multi-walled carbon nanotubes; PES: poly(ethylene succinate).

Table S2. Comparisons for non-activated AMEs of the calculated peak-to-peak separations (ΔE_p), heterogeneous electron transfer (k^0_{obs}) and electrochemically active area (A_e) for the filaments used in this work. Calculated using cyclic voltammetry (5-300 mV s^{-1}) in a solution of hexaamineruthenium (III) chloride (1 mM in 0.1 M KCl), with either 3D printed counter electrodes (CE) and reference electrodes (RE) or a nichrome wire CE and Ag|AgCl RE.

Filament	RE/CE	ΔE_p (V)	k^0_{obs} (cm s^{-1})	A_e (cm^2)
**Commercial PLA/CB**	External	0.122 ± 0.022	(1.83 ± 0.65) x 10^{-3}	0.105 ± 0.039
	3D-Printed	0.107 ± 0.047	(1.16 ± 0.32) x 10^{-3}	0.116 ± 0.049
**Recycled 8.78% PES**	External	0.138 ± 0.031	(1.63 ± 0.57) x 10^{-3}	0.139 ± 0.024
	3D-Printed	0.142 ± 0.025	(1.63 ± 0.76) x 10^{-3}	0.125 ± 0.036
**Recycled 10.98% PES**	External	0.118 ± 0.025	(1.96 ± 0.66) x 10^{-3}	0.126 ± 0.032
	3D-Printed	0.124 ± 0.039	(1.83 ± 0.48) x 10^{-3}	0.121 ± 0.037
**Recycled 13.15% PES**	External	0.159 ± 0.041	(1.27 ± 0.28) x 10^{-3}	0.143 ± 0.044
	3D-Printed	0.159 ± 0.041	(1.28 ± 0.29) x 10^{-3}	0.145 ± 0.060

Table S3. Peak potential shift between Ag|AgCl reference and 3D-printed *pseudo*-reference for both hexaamineruthenium (III) chloride (1 mM in 0.1 M KCl) and ferrocenemethanol (1 mM in 0.1 M KCl).

Filament	E_p shift (RuHex, mV)	E_p shift (FcMeOH, mV)
**Commercial PLA/CB**	84 ± 20	125 ± 10
**Recycled 8.78% PES**	84 ± 5	130 ± 7
**Recycled 10.98% PES**	97 ± 8	110 ± 5
**Recycled 13.15% PES**	83 ± 2	113 ± 4

Table S4. Comparison of the performance of the produced cell to other electroanalytical caffeine sensors in the literature.

Sensor	E_{pa} (V)	Technique	LDR (µM)	Sensitivity (µA µM^{-1})	LOD (µM)	Ref.
G-BCPE	+1.53[a]	SWV	2.0−800	1.83	0.147	[8]
BQMCPE	+1.45[a]	SWV	0.0−500	0.01	0.30	[9]
NCOMCP	+1.35[b]	DPV	5.0−20	0.19	0.016	[10]
3D CB-PLA	+1.5 and +1.4[a]	CV and DPV	10−1000	0.21 and 0.064	0.49 and 0.40	[11]
SWCNT/CCE	+1.38[b]	DPV	0.25−100	0.83	0.12	[12]
3D r-PLA/CB/10% PES	*+1.45*[c]	*DPV*	*1.0−500*	*0.055*	*0.23*	*This work*

[a] *vs.* Ag/AgCl; [b] *vs. SCE;* [c] *vs.* 3D *pseudo*-RE.

G-BCPE: Graphite Bare Carbon Paste Electrode; BQMCPE: 1,4-benzoquinone modified carbon paste electrode; NCOMCP: Nano-cobalt (II, III) oxide modified carbon paste electrode; 3D CB-PLA: Laser-treated conductive carbon black polylactic acid electrode; SWCNT/CCE: Single-walled carbon nanotubes on carbon-ceramic electrode.

8

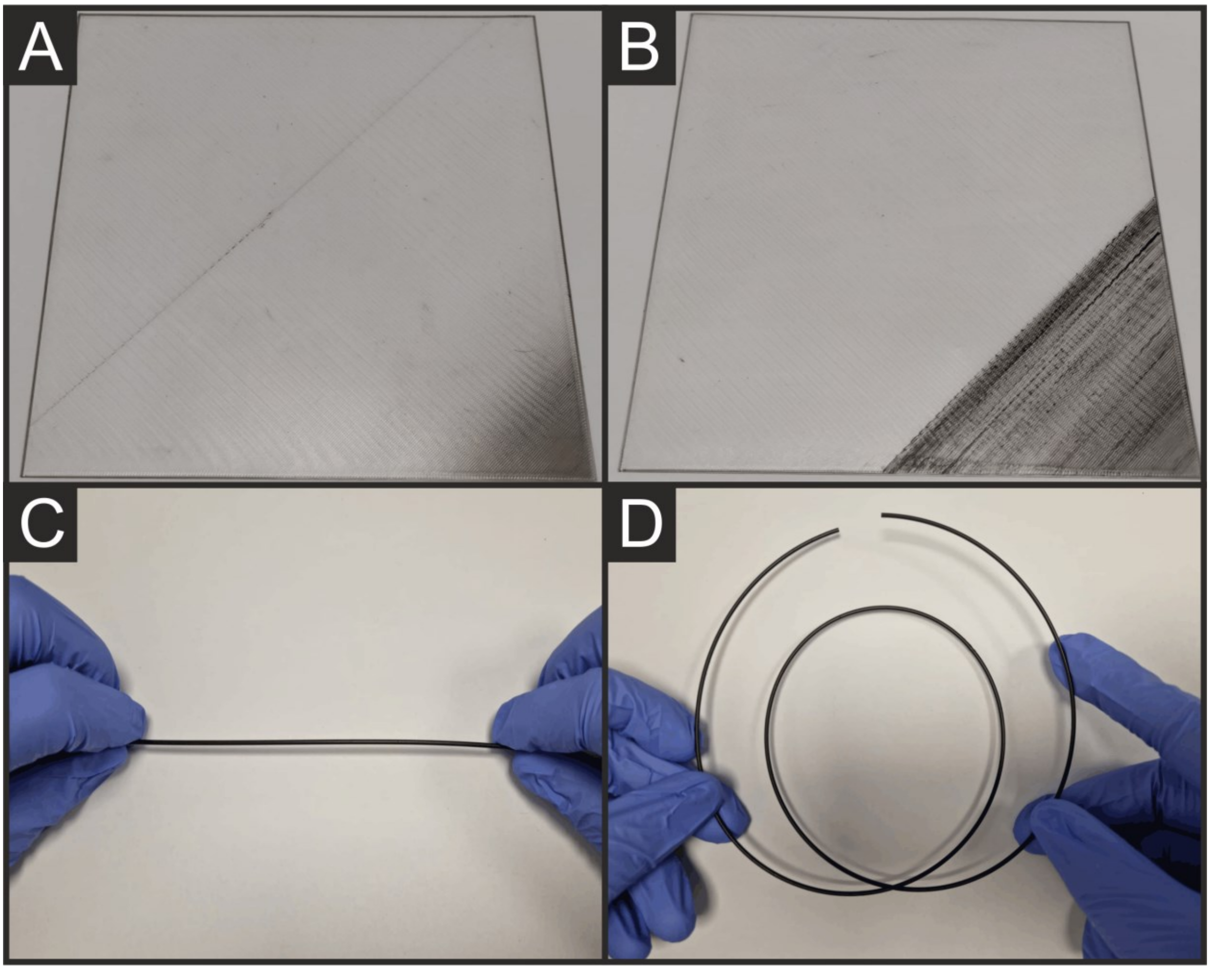

Figure S1. A comparison of the purging times between **A)** the 8.78% PES bespoke filament, and **B)** the commercial conductive filament. **C)** Photograph of the straight filament. **D)** Photograph of the bent filament, highlighting the flexibility.

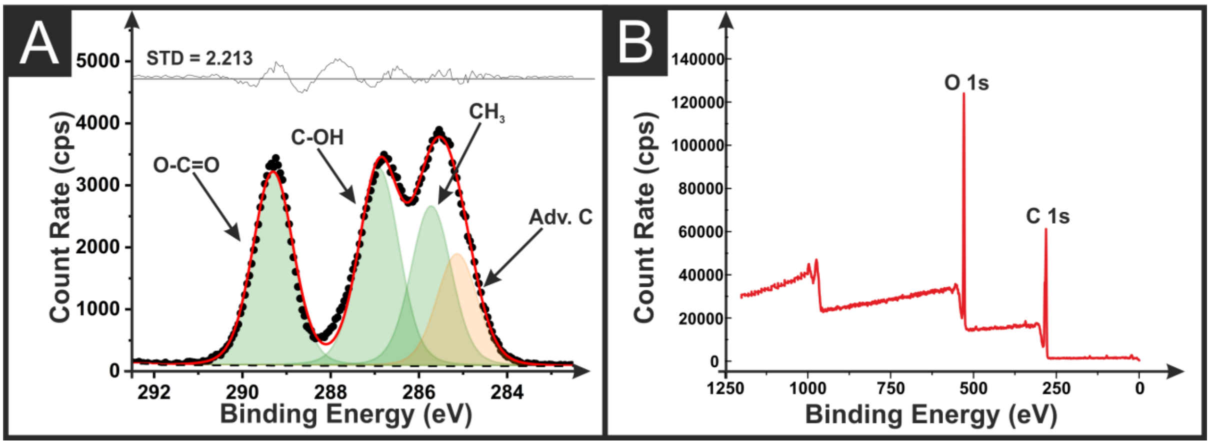

Figure S2. A) XPS C1s data for the non-activated 8.78% PES electrode. **B)** Wide angle XPS for the activated 8.78% PES electrode.

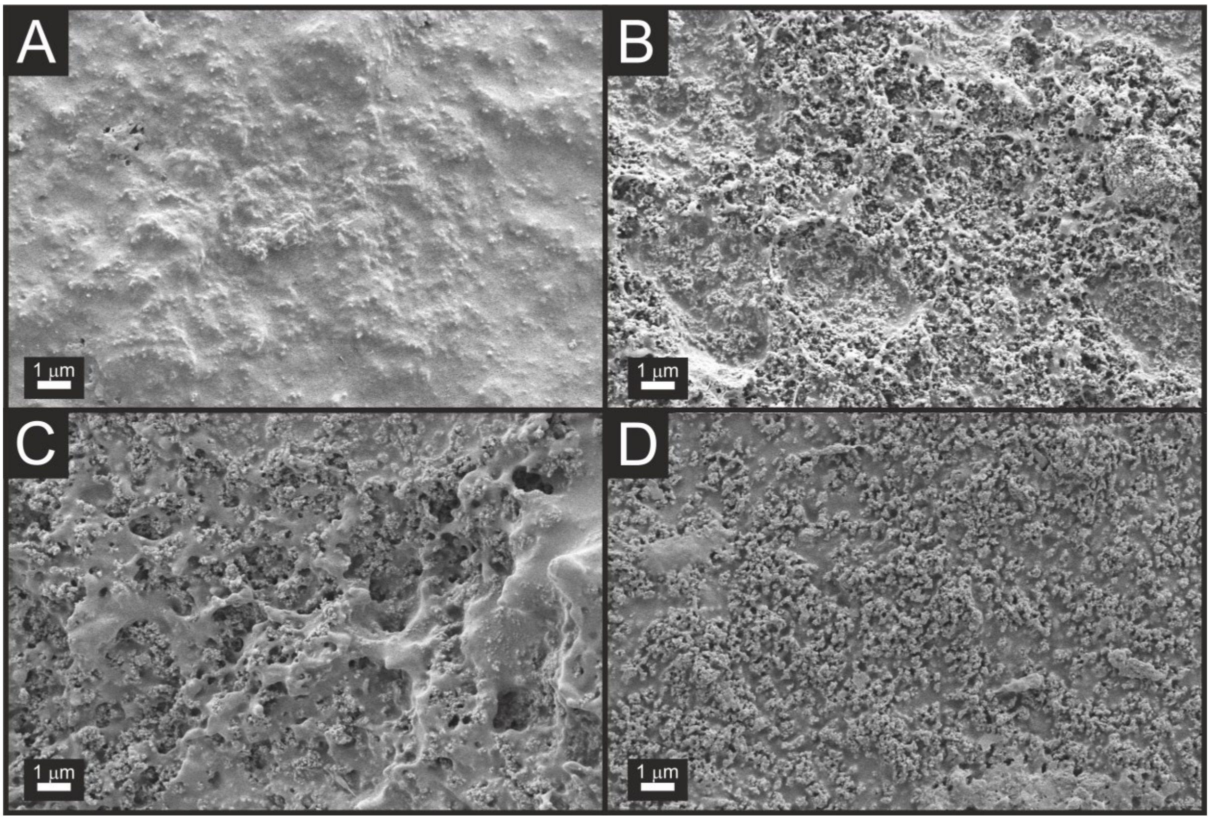

Figure S3. Scanning electron microscopy images for additively manufactured electrodes made from **A)** non-activated 8.78% PES, **B)** activated commercial PLA/CB filament, **C)** activated 10.98% PES filament, and **D)** activated 13.15% PES filament.

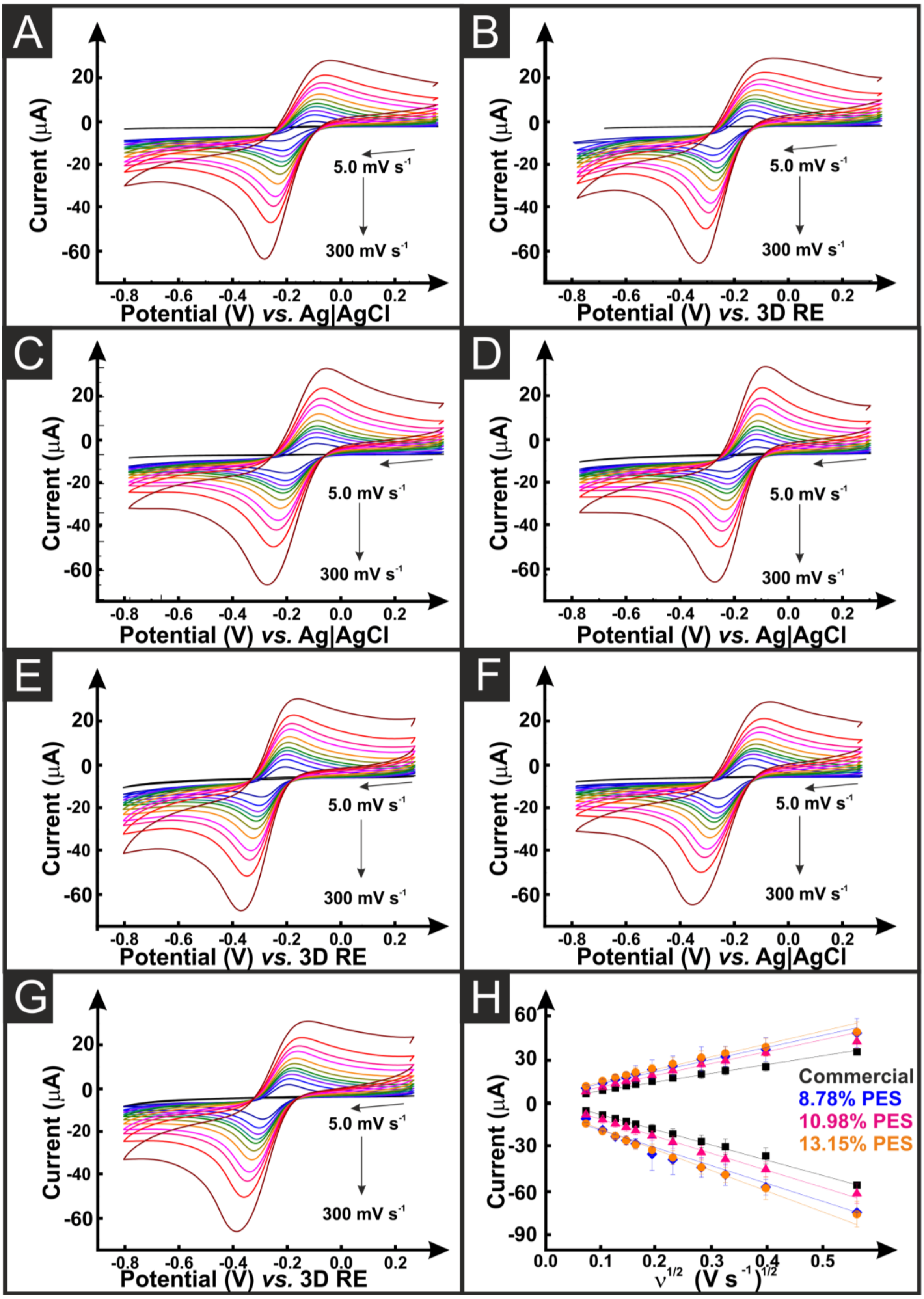

Figure S4. Cyclic voltammograms corresponding to the scan rate studies of 1 mM hexaamineruthenium (III) chloride in 0.1 M KCl using commercial AMEs with commercial **(A)** and printed **(B)** counter and references, 8.78% PES AME with a commercial **(C)** external and reference, 10.98% PES with commercial **(D)** and printed **(E)** counter and reference, and 13.15% PES AMEs with commercial **(F)** and printed **(G)** counter and references. **H)** Randle-Sevcik plot for the AMEs with commercial counter and reference electrodes.

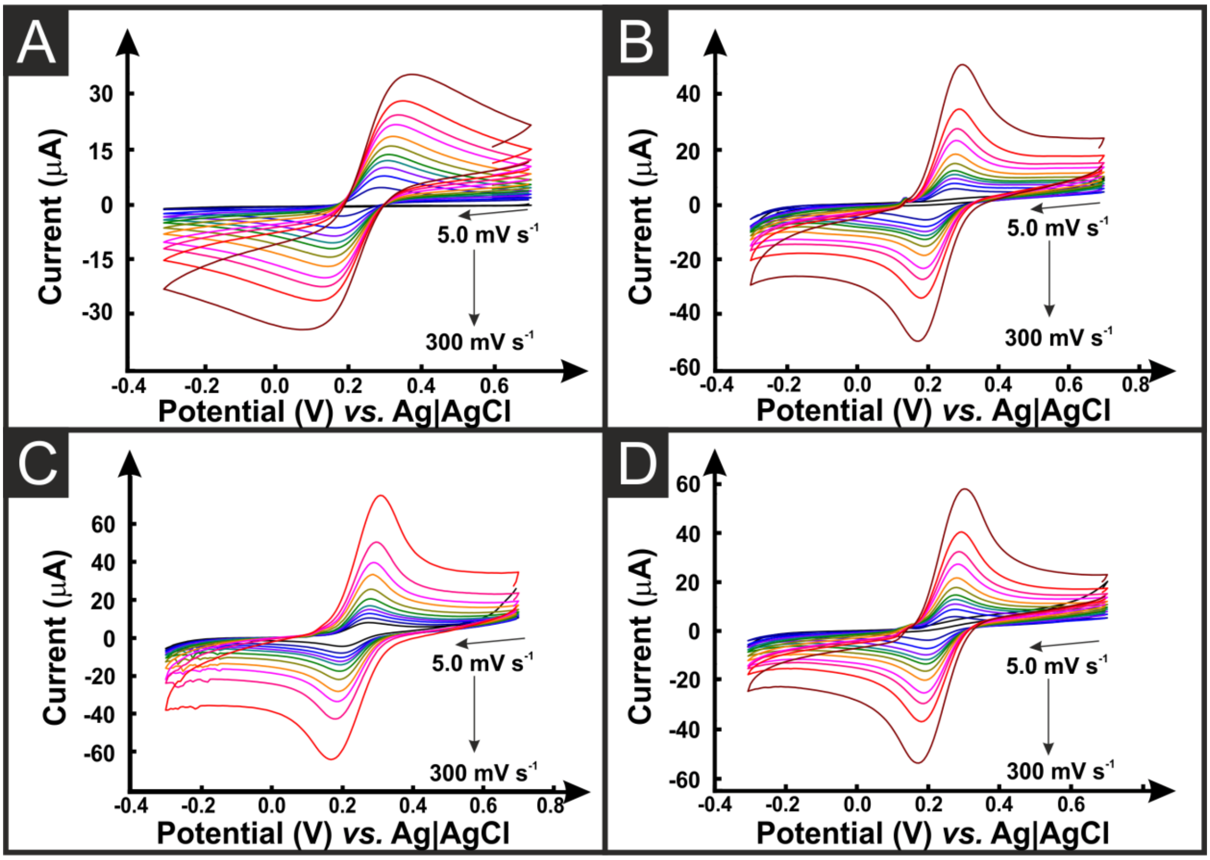

Figure S5. Cyclic voltammetric scan rate studies in ferrocene methanol (1 mM, 0.1 M KCl) using a commercial counter and reference electrode for **A)** Commercial AME, **B)** 8.78% PES AME, **C)** 10.98% PES AME, and **D)** 13.15% PES AME.

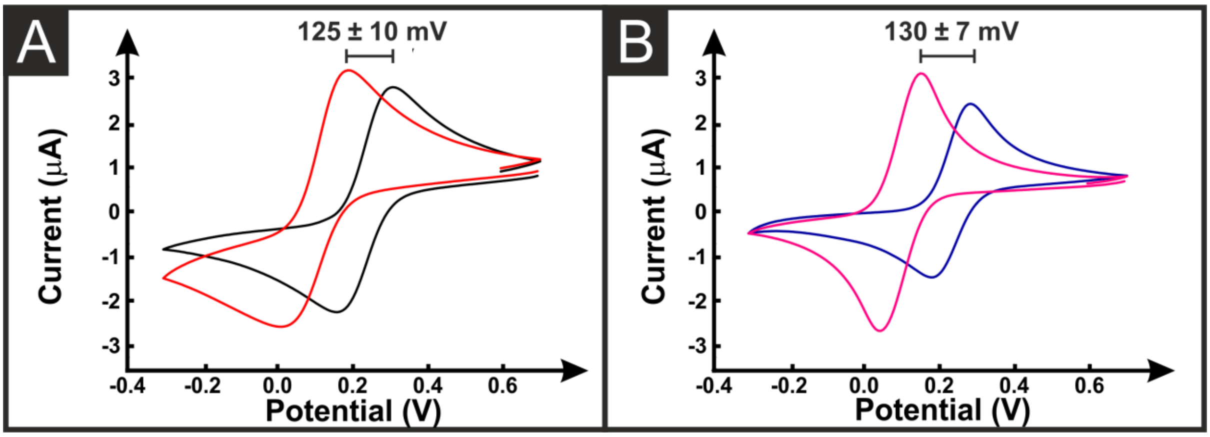

Figure S6. Cyclic voltammograms (25 mV s^{-1}) for ferrocene methanol (1 mM, 0.1 M KCl) showing the difference in peak position when using a commercial counter and reference compared to 3D-printed ones for **A)** AMEs printed from commercial filament **B)** AMEs printed from 8.78% PES recycled filament. Note in each case, the voltammogram to the left is using the printed counter and reference electrodes.

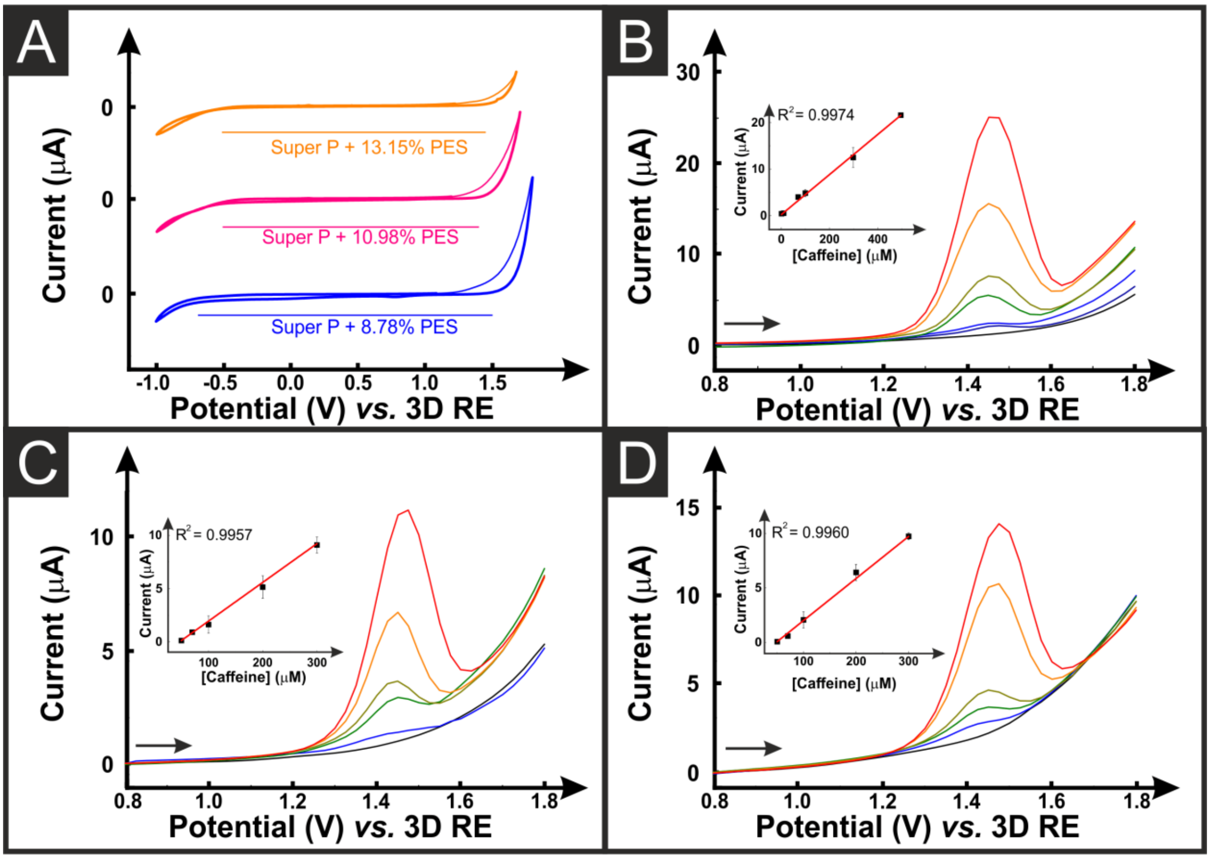

Figure S7. A) Cyclic voltammograms (25 mV s^{-1}) in 0.1 M phosphate buffer showing the potential window for the 8.78%, 10.98% and 13.15% PES filaments with AM counter and *pseudo*-reference electrodes. **B-D)** Differential pulse voltammograms of caffeine (pH 5.8) with **(B)** non-activated 8.78% PES **(C)** non-activated commercial and **(D)** activated commercial AMEs as the working electrodes and AM counter and *pseudo*-reference electrode. Inset is a plot of the peak current *versus* the concentration of caffeine.

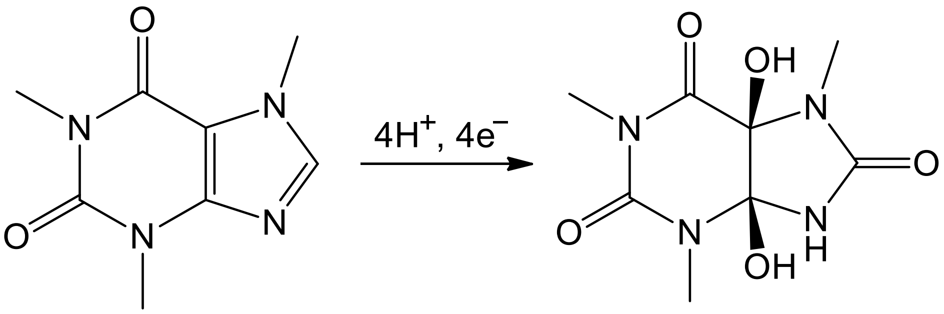

Figure S8. Irreversible oxidation reaction of caffeine to uric acid-4,5 diol.

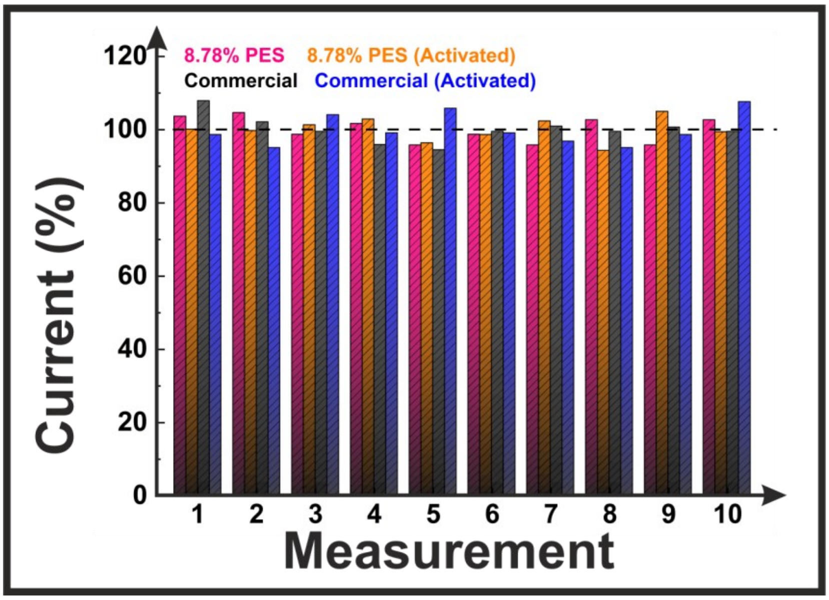

Figure S9. DPV measurements (n=10) showing the reproducibility of each electroanalytical cell. Performed in the presence of 100 µM caffeine using the non-activated and activated 8.78% PES (RSD = 3.48% and 3.14%) and commercial AMEs (RSD = 4.37% and 3.60%) as the working electrodes and AM counter and *pseudo*-reference electrode.

9

References:

[1] C.W. Foster, G.Q. Zou, Y. Jiang, M.P. Down, C.M. Liauw, A. Garcia-Miranda Ferrari, X. Ji, G.C. Smith, P.J. Kelly, C.E. Banks, Next-Generation Additive Manufacturing: Tailorable Graphene/Polylactic (acid) Filaments Allow the Fabrication of 3D Printable Porous Anodes for Utilisation within Lithium-Ion Batteries, Batteries & Supercaps 2(5) (2019) 448-453.

[2] J.S. Stefano, L.R.G. e Silva, R.G. Rocha, L.C. Brazaca, E.M. Richter, R.A.A. Muñoz, B.C. Janegitz, New conductive filament ready-to-use for 3D-printing electrochemical (bio) sensors: towards the detection of SARS-CoV-2, Analytica Chimica Acta 1191 (2022) 339372.

[3] S.W. Kwok, K.H.H. Goh, Z.D. Tan, S.T.M. Tan, W.W. Tjiu, J.Y. Soh, Z.J.G. Ng, Y.Z. Chan, H.K. Hui, K.E.J. Goh, Electrically conductive filament for 3D-printed circuits and sensors, Applied Materials Today 9 (2017) 167-175.

[4] K. Ghosh, S. Ng, C. Iffelsberger, M. Pumera, 2D MoS2/carbon/polylactic acid filament for 3D printing: Photo and electrochemical energy conversion and storage, Applied Materials Today 26 (2022) 101301.

[5] M.P. Down, E. Martínez-Periñán, C.W. Foster, E. Lorenzo, G.C. Smith, C.E. Banks, Next-generation additive manufacturing of complete standalone sodium-ion energy storage architectures, Advanced Energy Materials 9(11) (2019) 1803019.

[6] C.W. Foster, H.M. Elbardisy, M.P. Down, E.M. Keefe, G.C. Smith, C.E. Banks, Additively manufactured graphitic electrochemical sensing platforms, Chemical Engineering Journal 381 (2020) 122343.

[7] J.P. Hughes, P.L. dos Santos, M.P. Down, C.W. Foster, J.A. Bonacin, E.M. Keefe, S.J. Rowley-Neale, C.E. Banks, Single step additive manufacturing (3D printing) of electrocatalytic anodes and cathodes for efficient water splitting, Sustainable Energy & Fuels 4(1) (2020) 302-311.

[8] Y. Tadesse, A. Tadese, R. Saini, R. Pal, Cyclic voltammetric investigation of caffeine at anthraquinone modified carbon paste electrode, International Journal of Electrochemistry 2013 (2013).

[9] M. Aklilu, M. Tessema, M. Redi-Abshiro, Indirect voltammetric determination of caffeine content in coffee using 1, 4-benzoquinone modified carbon paste electrode, Talanta 76(4) (2008) 742-746.

[10] A. Fekry, M. Shehata, S. Azab, A. Walcarius, Voltammetric detection of caffeine in pharmacological and beverages samples based on simple nano-Co (II, III) oxide modified carbon paste electrode in aqueous and micellar media, Sensors and Actuators B: Chemical 302 (2020) 127172.

[11] M.J. Głowacki, M. Cieślik, M. Sawczak, A. Koterwa, I. Kaczmarzyk, R. Jendrzejewski, Ł. Szynkiewicz, T. Ossowski, R. Bogdanowicz, P. Niedziałkowski, Helium-assisted, solvent-free electro-activation of 3D printed conductive carbon-polylactide electrodes, Applied Surface Science 556 (2021) 149788.

[12] B. Habibi, M. Abazari, M.H. Pournaghi-Azar, A carbon nanotube modified electrode for determination of caffeine by differential pulse voltammetry, Chinese Journal of Catalysis 33(11-12) (2012) 1783-1790.
